# Synchronization of complex networks of identical and nonidentical chaotic systems via model-matching control

**DOI:** 10.1371/journal.pone.0216349

**Published:** 2019-05-23

**Authors:** D. López-Mancilla, G. López-Cahuich, C. Posadas-Castillo, C. E. Castañeda, J. H. García-López, J. L. Vázquez-Gutiérrez, E. Tlelo-Cuautle

**Affiliations:** 1 Centro Universitario de los Lagos, Universidad de Guadalajara (UDG), Jalisco, México; 2 Universidad Autónoma de Nuevo León (UANL), Nuevo León, México; 3 Instituto Nacional de Astrofísica Óptica y Electrónica (INAOE), Puebla, México; Lanzhou University of Technology, CHINA

## Abstract

In this work, a synchronization scheme for networks of complex systems is presented. The proposed synchronization scheme uses a control law obtained with some definitions from graph theory and solving the Model-Matching Problem for complex networks. In particular, Rössler, Chen, Lorenz and Lü chaotic systems are used as complex chaotic systems into complex networks. Particular cases with regular and irregular networks of six identical chaotic systems are implemented, with some well-known topologies as star and ring small-world, and tree topologies. Highlighting, the obtained control law is applied to synchronize an irregular network of six different chaotic systems in a tree topology. The usefulness and advantages of the proposed synchronization scheme are highlighted performing numerical simulations of the chaotic complex networks.

## Introduction

The field of synchronization of networks of complex systems has received a lot of attention in the last three decades, due to the potential applications in engineering, the proliferation of computer networks, communications networks as the internet, wireless communications as cellular telephony and many others [1, 2]. Different methods and topologies have been proposed for network synchronization. For example, the authors in [3–6] introduced schemes for the synchronization in small-world dynamical networks. Serrano and co-workers made a contribution on chaotic synchronization in star coupled neural networks [7]. Model-Matching Control (MMC) has been used as a solution for some open problems. It has been recently used for output synchronization of heterogeneous and nonlinear multi-agent systems [8, 9]. In other work, the authors consider the model matching problem for a class of multiple-input systems whose inputs involve different delays [10]. In chaos synchronization area, MMC has been used in the last years, but only to synchronize pairs of identical and nonidentical chaotic oscillators [11–13]. Chaotic synchronization of regular and irregular complex networks with fractional order oscillators has been presented in [14]. Nevertheless, most of the authors show results in synchronization for only structurally identical complex systems, whereas for synchronization of nonidentical complex systems, the results are only a few. Synchronization, output-synchronization, partial-synchronization or phase-synchronization of nonidentical complex systems would have implication to understand different areas. For example in neural networks, the interaction between pre-synaptic and post-synaptic neurons, where the pre-synaptic neurons output activates the nucleus cells in post-synaptic neurons to achieve some kind of synchronization between two different neurons [15]. In communications systems, sometimes the network topology has different complex systems and it is required to synchronize at least the nodes outputs. This is necessary to encryption/decryption and transmission of information [16].

The aim of this work is also devoted to demonstrate the effectiveness of the MMC for synchronizing networks of complex/chaotic systems in continuous-time. This objective is achieved by using the model-matching approach from nonlinear control theory [17, 18] and extending the results in [11, 12]. The main contribution is the generalization of a MMC for network synchronization and highlight that the proposed scheme has the following advantages:

- It is a systematic procedure to be used as guideline or a kind of recipe that anyone can reproduce.
- It can be useful to synchronize networks of identical and nonidentical complex/chaotic systems.
- It can be used in unidirectional and bidirectional coupling between master and slave systems.
- It can be used to synchronizing chaotic and hyperchaotic systems [19].
- Any node can be chosen as a master node.
- There may be more than one master node.

The proposed MMC is tested to synchronize networks of different chaotic systems, like: Rössler, Chen, Lorenz and Lü chaotic oscillators. Two classic regular network topologies are taken as cases of study, namely a star topology with an isolated node, and a ring topology. In the same way, two irregular topologies are considered, such as:: a small-world topology and a tree topology. All topologies use six chaotic systems as nodes. The election of using six nodes can be extended to larger networks. Nevertheless, it is well known that if the number of the nodes is big enough, then complete synchronization could be guaranteed only for some topologies [20]. Readers interested in pattern formation in complex networks and some new results see [21–24].

This work is organized as follows: Section 2 states the problem formulation for synchronizing a network of chaotic systems. The model-matching problem from nonlinear control theory applied to network synchronization is presented in Section 3. In Section 4, this approach is applied to synchronize networks of identical and nonidentical chaotic systems based on Rössler, Lorenz, Lü and Chen systems, with different topologies. Finally, Section 5 summarizes the concluding remarks.

## Problem statement

Consider a network of complex dynamical systems described by state equations of the form:

$$\begin{array}{c} P_{i}:\{\begin{array}{c} {\dot{x}}_{i}=f(x_{i})+g(x_{i})u_{i},\\ y_{i}=h(x_{i}), \end{array} \end{array}$$ (1)

where the states $x_{i}\;(t)\in R^{n},$ the inputs $u_{i}\;(t)\in R,$ and the outputs $y_{i}\;(t)\in R$, being *f* (*x*_*i*_) and *g* (*x*_*i*_) smooth and analytical functions and with *i* = 1, 2, …, *N*, where *N* is the number of nodes in the network. In addition, consider another nonlinear system described by:

$$\begin{array}{c} M:\{\begin{array}{c} {\dot{x}}_{M}=f_{M}(x_{M})+g_{M}(x_{M})u_{M},\\ y_{M}=h_{M}(x_{M}), \end{array} \end{array}$$ (2)

where the state $x_{M}\;(t)\in R^{n_{M}},$ the input $u_{M}\;(t)\in R,$ and the output $y_{M}\;(t)\in R$, being *f*_*M*_ (*x*_*M*_) and *g*_*M*_ (*x*_*M*_) also smooth and analytical functions. Assume that $x_{i}^{{}^{\circ}}$ are equilibrium points of systems described in (1), i.e., $f(x_{i}^{{}^{\circ}})=0$. Similarly, $x_{M}^{{}^{\circ}}$ is an equilibrium point of system (2). Assume that the dynamical systems of (1) and (2) under certain conditions have *chaotic* behavior. Then, the chaotic system (1) *synchronizes* with the chaotic system (2), if:

$$\begin{array}{c} \lim_{t\to \infty }|y_{i}(t)-y_{M}(t)|=0, \end{array}$$ (3)

no matter which initial conditions *x*_*i*_ (0) and *x*_*M*_ (0) have, and for each node *i* = 1, 2, …, *N* and suitable input signals *u*_*i*_ (*t*) and *u*_*M*_ (*t*).

Note that, it is mainly considering **output synchronization problem** between a network of chaotic systems (1) and (2). Moreover, no matter if the chaotic systems (1) and (2) are identical or nonidentical. In the next section, it will be described how to satisfy the output synchronization condition (3) from the perspective of the model-matching problem and graph theory.

## Model-matching for networks

Let us consider the dynamical systems (1) like *plants P*_*i*_, and (2) like a *model* *M*, respectively. The objective is to generalize a feedback control law *u* (*t*) for several plants *P*_*i*_ which, irrespectively of the initial states of *P*_*i*_ and *M*, makes the outputs *y*_*i*_ (*t*) converge asymptotically to the output *y*_*M*_ (*t*) produced by *M* under an arbitrary input *u*_*M*_ (*t*). This problem is the called asymptotic *model-matching problem* from nonlinear control theory, and it was solved for synchronizing only a pair of chaotic systems in [11, 12]. Previously, different approaches to solve the model-matching problem have been proposed in the literature, see e.g. [17, 18]. In this work, the solution proposed in [11, 12] is extended to synchronize several chaotic plants with one chaotic model. In the design of the MMC we considered the possibility of using more than one chaotic model into a network of complex chaotic systems. Then, for network synchronization purpose, *N auxiliary systems* are defined by the following equation:

$$\begin{array}{c} E_{i}:\{\begin{array}{c} {\dot{x}}_{Ei}=f_{E}(x_{Ei})+\hat{g}(x_{Ei})u_{i}+{\hat{g}}_{M}(x_{Ei})u_{M},\\ y_{Ei}=h_{E}(x_{Ei}), \end{array} \end{array}$$ (4)

with state $x_{Ei}=(x_{i},x_{M}{)}^{⊤}\in R^{n+n_{M}}$, inputs *u* (*t*) and *u*_*M*_ (*t*), and

$$\begin{array}{ccc} f_{E}\left(x_{Ei}\right) & =\left(\begin{array}{c} f\left(x_{i}\right)\\ f_{M}\left(x_{M}\right) \end{array}\right), & \hat{g}\left(x_{Ei}\right)=\left(\begin{array}{c} g\left(x_{i}\right)\\ 0 \end{array}\right),\\ {\hat{g}}_{M}\left(x_{Ei}\right) & =\left(\begin{array}{c} 0\\ g_{M}\left(x_{M}\right) \end{array}\right), & h_{E}\left(x_{Ei}\right)=h\left(x_{i}\right)-h_{M}\left(x_{M}\right). \end{array}$$

Note that the output *y*_*Ei*_ = *h*_*E*_ (*x*_*Ei*_) of the auxiliary system (4) is the difference between the output of the plant *P*_*i*_ [*y*_*i*_ = *h* (*x*_*i*_) of Eq (1)] and the output of system *M* [*y*_*M*_ = *h*_*M*_ (*x*_*M*_) of Eq (2)]. The control objective of the model-matching problem is contained in the following definition:

**Definition 1 (Model-matching problem)**: *Given the plants P_i_ and the model M around their respective equilibrium points* $x_{i}^{{}^{\circ}}$ *and* $x_{M}^{{}^{\circ}},$ *and points* $x_{Ei}^{{}^{\circ}}$. *The model-matching problem consists of finding feedback control laws* $u_{i}(t)\in R$ *for the auxiliary system E*_*i*_ (4) *such that, the output y*_*Ei*_ (*t*) → 0 *as t* → ∞.

The equilibrium points in Definition 1, are stated in the following Definition:

**Definition 2 (Relative degree adapted from** [18]): *The single-input single-output nonlinear system* (1), *is said to have* **relative degree** *r*_*i*_ *at points* $x_{i}^{{}^{\circ}}$ *if*:

1. $L_{g}L_{f}^{k}h(x_{i})=0$ *for all x*_*i*_ *in a neighborhood of* $x_{i}^{{}^{\circ}}$ *and for all k* < *r*_*i*_ − 1,
2. $L_{g}L_{f}^{r_{i}-1}h(x_{i}^{{}^{\circ}})\neq 0$.

In Definition 2, $L_{f}h(x_{i})=\frac{\partial h(x_{i})}{\partial x_{i}}f(x_{i})$ and $L_{g}L_{f}^{k}h(x_{i})=\frac{\partial (L_{f}^{k}h(x_{i}))}{\partial x_{i}}g(x_{i})$. A similar definition can be given for the relative degree of model (2), *r*_*M*_ near $x_{M}^{{}^{\circ}}$. It is important to mention that the model matching problem is locally solvable if, and only if [18]:

$$\begin{array}{c} r_{i}\leq r_{M}. \end{array}$$ (5)

Now, let the auxiliary system *E*_*i*_ Eq (4) be in a different coordinate frame. From definition of relative degrees *r*_*i*_ and *r*_*M*_; $h\;(x_{i}),\ldots ,L_{f}^{n-1}h\;(x_{i}),$ and $h_{M}\;(x_{M}),\ldots ,L_{f_{M}}^{n-1}h_{M}\;(x_{M})$ are sets of independent functions from *P*_*i*_ and *M*, and can be chosen as new coordinates $\xi_{q}\;(x_{i})=L_{f}^{q-1}h\;(x_{i})$ and $\xi_{Mq}\;(x_{M})=L_{f_{M}}^{q-1}h_{M}\;(x_{M})$ with *q* = 1, …, *n*, around $x_{i}^{{}^{\circ}}$ and $x_{M}^{{}^{\circ}}$, respectively. Consider now the auxiliary system *E*_*i*_ and the new coordinates [18]:

$$\begin{array}{c} (\zeta (x_{Ei}),x_{M})=\phi (x_{Ei})=\phi (x_{i},x_{M}), \end{array}$$

where *ζ* (*x*_*Ei*_) = (*ζ*_1,*i*_ (*x*_*Ei*_), …, *ζ*_*n*,*i*_ (*x*_*Ei*_))^⊤^, and $\zeta_{q,i}\;(x_{Ei})=L_{f_{Ei}}^{q-1}h_{Ei}\;(x_{Ei})=\xi_{q,i}\;(x_{i})-\xi_{Mq}\;(x_{M}),$ *q* = 1, …, *n*.

Thus, the closed-loop auxiliary system *E*_*i*_, using the following feedback control law

$$\begin{array}{c} u_{i}=\frac{1}{L_{g}L_{f}^{n-1}h(x_{i})}(v_{i}-L_{f}^{n}h(x_{i})+L_{f_{M}}^{n}h_{M}(x_{M})+\;L_{g_{M}}L_{f_{M}}^{n-1}h_{M}(x_{M})u_{M}), \end{array}$$ (6)

takes the form:

$$\begin{array}{c} \begin{array}{cc} {\dot{\zeta }}_{q,i} & =\zeta_{q+1,i},\;\;\;\;\;\;q=1,\ldots ,n-1,\;\;\;\;\;\;i=1,\ldots ,N,\\ {\dot{\zeta }}_{n,i} & =v_{i}=-c_{0,i}\;\zeta_{1,i}-\;\ldots \;-c_{n,i}\;\zeta_{n,i},\\ {\dot{x}}_{M} & =f_{M}(x_{M})+g_{M}(x_{M})u_{M},\\ y_{Ei} & =\zeta_{1,i}. \end{array} \end{array}$$ (7)

Two subsystems can be identified in the closed-loop system (7), namely:

1. The subsystem described by:
  $$\begin{array}{c} {\dot{x}}_{M}=f_{M}(x_{M})+g_{M}(x_{M})u_{M}, \end{array}$$
  which represents the dynamics of *M*, and
2. The subsystem described by:
  $$\begin{array}{c} \dot{\zeta_{i}}=A_{i}^{*}\zeta_{i}, \end{array}$$
  with
  $$\begin{array}{c} A_{i}^{*}=[\begin{array}{ccccc} 0 & 1 & 0 & \ldots & 0\\ 0 & 0 & 1 & \ldots & 0\\ \vdots & \vdots & \vdots & \ddots & \vdots \\ 0 & 0 & 0 & \ldots & 1\\ -c_{0,i} & -c_{1,i} & -c_{2,i} & \ldots & -c_{n-1,i} \end{array}], \end{array}$$
  which represents the dynamics of *y*_*Ei*_ (*t*). The matrix $A_{i}^{*}$ can be a single constant matrix choosing *i* = 1 or it can define different matrix, specially when they are used for synchronization of non-identical chaotic systems into a complex network.

Model *M* is stable by assumption, since it is proposed, and the control law *v*_*i*_ (*t*) can be selected such that eigenvalues of matrix A* have a negative real part. Then the closed-loop system will be exponentially stable, and output synchronization condition (3) holds.

### Complex networks and model-matching

From a mathematical point of view, a complex network is defined as an interconnected set of a nodes (two or more) and can be represented by a graph, where two nodes (vertices or points) joined by a connection (edges or lines) are called adjacent nodes or neighbors. Some of the most notable features for complex systems are:

- They consist of many interacting parts (nodes).
- Each part has its own internal structure and is responsible for a specific task.

Topology or coupling mesh is the layout or how the nodes of a network are connected, while the configuration is the type of connection that determines the flow of information between nodes. Now, consider the traditional control law for network synchronization [3, 14]

$$\begin{array}{c} u_{i1}=c\sum_{j=1}^{N}a_{i,j}\Gamma x_{j},\;\;\;\;\;\;i=1,\ldots ,N, \end{array}$$

where *N* denotes the size of the network or the number of nodes, *c* > 0 represents the coupling strength, and $\Gamma \in R^{n\times n}$ is a constant matrix linking the state variables. The matrix $A=\left(a_{i,j}\right)\in R^{n\times n}$ is the coupling matrix. If there is a connection between nodes *i* and *j* then, the element *a*_*i*,*j*_ = 1; otherwise *a*_*i*,*j*_ = 0, *i* ≠ *j*. For *i* = *j* the diagonal elements of *A* are defined as:

$$\begin{array}{c} a_{i,i}=-\sum_{j=1,j\neq i}^{N}a_{i,j}=-\sum_{j=1,j\neq i}^{N}a_{j,i},\;\;\;\;\;\;i=1,\ldots ,N. \end{array}$$

Then, given the previous result and the above definitions, it is possible to formulate a general control law for network synchronization using Model-Matching control, and choosing *r*_*i*_ = *r*_*M*_ = *r*, as:

$$\begin{array}{c} u_{i}=\sum_{j=1}^{N}a_{i,j}u_{i,j}\;\;\;\;\;\;i=1,\ldots ,N, \end{array}$$

This is:

$$\begin{array}{c} \begin{array}{c} u_{1}=a_{1,1}u_{1,1}+a_{1,2}u_{1,2}+\cdots +a_{1,N}u_{1,N}\\ u_{2}=a_{2,1}u_{2,1}+a_{2,2}u_{2,2}+\cdots +a_{2,N}u_{2,N}\\ \vdots \\ u_{N}=a_{N,1}u_{N,1}+a_{N,2}u_{N,2}+\cdots +a_{N,N}u_{N,N} \end{array} \end{array}$$

and

$$\begin{array}{c} u_{i,j}=\frac{1}{L_{g}L_{f}^{r-1}h(x_{i})}\left\{v_{i,j}-L_{f}^{r}h(x_{i})+L_{f}^{r}h(x_{j})+\;L_{g}L_{f}^{r-1}h(x_{j})u_{j}\right)\}, \end{array}$$

where *x*_*i*_ and *x*_*j*_ represents the states of the chaotic systems nodes, plants and models, respectively. Notice that with this control law, any node can be chosen as the model system *M*.

## Network synchronization through model-matching control for identical and nonidentidal systems

**Fig 1:**
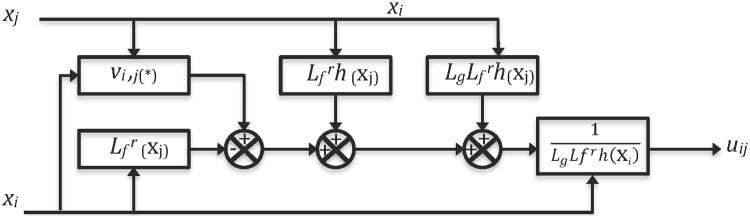
Block diagram of model-matching control.

**Fig 2:**
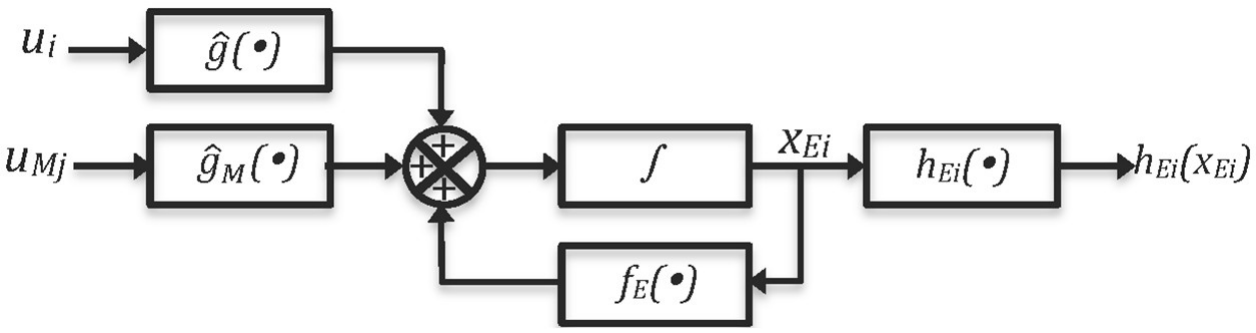
Auxiliary system block diagram.

In this section, we use the previous material in order to illustrate how network synchronization of *N* chaotic systems can be achieved. Two cases of study are considered, using identical and nonidentical chaotic systems like nodes in regular and irregular topologies. The number of chaotic nodes are limited to *N* = 6 nodes. Fig 1 shows the block diagram of model-matching control and Fig 2 presents the auxiliary system block diagram. The model matching control works as a similar way like previous works from one of the authors, but adapted to network synchronization [11, 12]. Thus, for identical chaotic systems, complete synchronization is guaranteed for a relative degree *r* = *n*. For nonidentical chaotic systems only output synchronization is guaranteed.

Table 1 presents the equations of the chaotic systems and the parameter values for Rössler, Chen, Lorenz and Lü chaotic systems, used to illustrate synchronization using Model-Matching Control. Although the proposed approach can be applied to any chaotic system that holds (5) and for all plant *P*_*i*_ with a strong relative degree. In Table 1, Rössler systems have complete strong relative degree *r* = 3, the rest of the systems have a strong relative degree *r* = 2. For synchronization of identical systems with relative degree *r* = 2, stability demonstration is not trivial. But, the first two equations of the auxiliary system become linear and asymptotically stable, and the last one presents a zero dynamic isolated from the output, depending only from the third state, obtaining the form $\dot{\zeta_{3}}=-\beta \zeta_{3}$, for some positive constant *β*. Then, the solution *ζ*_3_ = *exp* (−*βt*) is exponentially stable.

**Table 1:** Chaotic systems and parameter values.

System	Equations	Parameter Values
Rössler	$\begin{array}{rr} \left(\begin{array}{c} {\dot{x}}_{i1}\\ {\dot{x}}_{i2}\\ {\dot{x}}_{i3} \end{array}\right) & =\left(\begin{array}{c} -x_{i2}-x_{i3}\\ x_{i1}+\alpha x_{i2}\\ \alpha +x_{i3}\left(x_{i1}-\mu \right) \end{array}\right)+\left(\begin{array}{l} 0\\ 0\\ 1 \end{array}\right)u\\ y_{i} & =x_{i2}. \end{array}$	*α* = 0.2, *μ* = 0.7.
Chen	$\begin{array}{rr} \left(\begin{array}{c} {\dot{x}}_{i1}\\ {\dot{x}}_{i2}\\ {\dot{x}}_{i3} \end{array}\right) & =\left(\begin{array}{c} \theta_{1}\left(x_{i2}-x_{i1}\right)\\ \left(\theta_{2}-\theta_{1}\right)x_{i1}-x_{i1}x_{i3}+\theta_{2}x_{i2}\\ x_{i1}x_{i2}-\theta_{3}x_{i3} \end{array}\right)+\left(\begin{array}{l} 0\\ 1\\ 0 \end{array}\right)u\\ y_{i} & =x_{i1}. \end{array}$	*θ*_1_ = 35, *θ*_2_ = 28, *θ*_3_ = 3.
Lorenz	$\begin{array}{rr} \left(\begin{array}{c} {\dot{x}}_{i1}\\ {\dot{x}}_{i2}\\ {\dot{x}}_{i3} \end{array}\right) & =\left(\begin{array}{c} \sigma \left(x_{i2}-x_{i1}\right)\\ rx_{i1}-x_{i2}-x_{i1}x_{i3}\\ x_{i1}x_{i2}-bx_{i3} \end{array}\right)+\left(\begin{array}{l} 0\\ 1\\ 0 \end{array}\right)u\\ y_{i} & =x_{i1}. \end{array}$	*σ* = 10, *r* = 28, *b* = 8/3.
Lü	$\begin{array}{rr} \left(\begin{array}{c} {\dot{x}}_{i1}\\ {\dot{x}}_{i2}\\ {\dot{x}}_{i3} \end{array}\right) & =\left(\begin{array}{c} a\left(x_{i2}-x_{i1}\right)\\ -x_{i1}x_{i3}+cx_{i2}\\ x_{i1}x_{i2}-bx_{i3} \end{array}\right)+\left(\begin{array}{l} 0\\ 1\\ 0 \end{array}\right)u\\ y_{i} & =x_{i1}. \end{array}$	*a* = 36, *b* = 28, *c* = 20.

### Regular networks

Some classic regular networks are used to illustrate the synchronization of networks of chaotic systems. In particular, Star topology and Ring topology are presented.

#### Star topology with an isolated node

**Fig 3:**
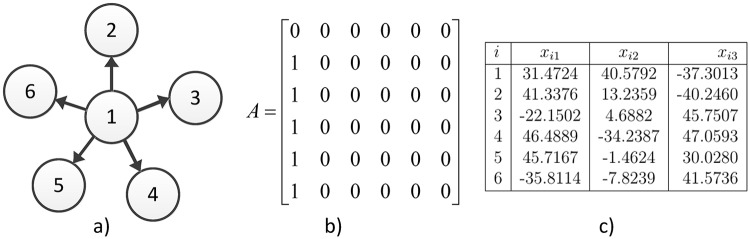
a) Star topology, b) coupling matrix, c) random initial conditions.

**Fig 4:**
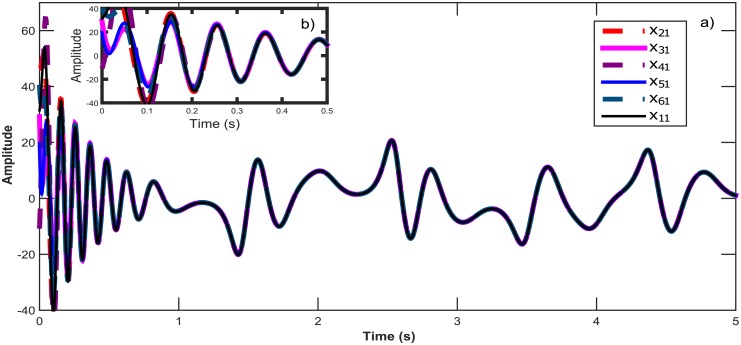
a) Temporal signals for the synchronization of six Chen chaotic systems in a star topology with an isolated node. b) Zoom in window showing the convergence in time for all *x*_*i*1_ states.

**Fig 5:**
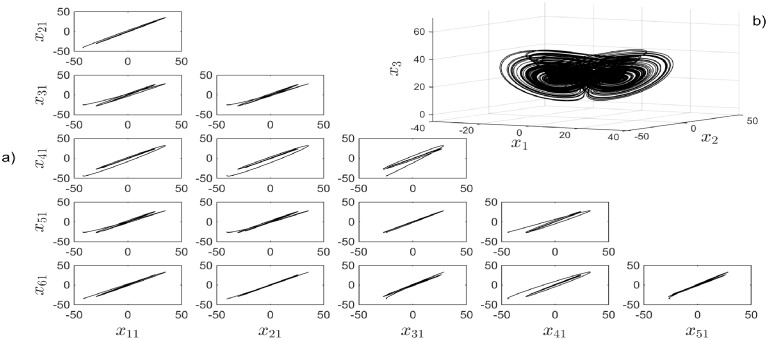
a) Synchronization graphics for six Chen chaotic systems in a star topology with an isolated node. b) Chen chaotic attractor.

Consider a network in a Star topology with six Chen hyperchaotic systems. Fig 3 shows the Star topology for six hyperchaotic systems and the associated coupling matrix. The temporal signals of six Chen hyperchaotic systems synchronizing are illustrated in Fig 4, whereas in Fig 5 the synchronization graphics for the outputs of six Chen hyperchaotic systems are presented. See [19] for discrete-time context.

#### Ring topology

**Fig 6:**
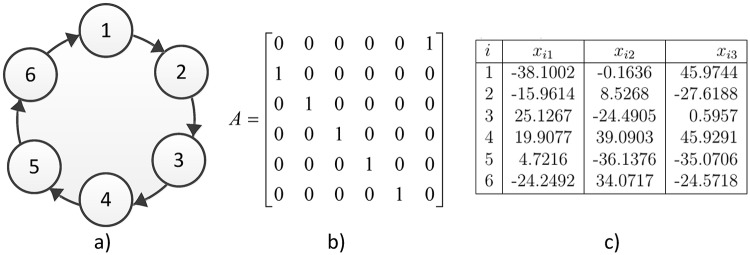
a) Ring topology b) coupling matrix, c) random initial conditions.

**Fig 7:**
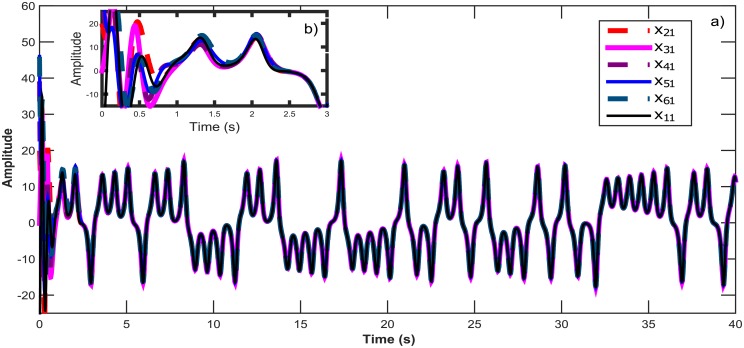
a) Temporal signal on the synchronization for six Lorenz chaotic systems in a ring topology. b) Zoom in window showing the convergence in time for all *x*_*i*1_ states.

**Fig 8:**
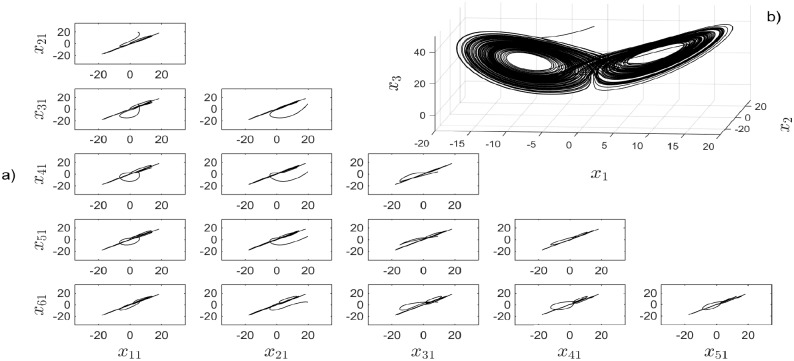
a) Synchronization graphics for six Lorenz chaotic systems in a ring topology with an isolated node. b) Lorenz chaotic attractor.

The second case is a Ring topology with six Lorenz systems shown in Fig 6 and its associated coupling matrix. Fig 7 illustrates the temporal signals on the synchronization of six Lorenz systems in a Ring topology and Fig 8 shows the synchronization graphics for the outputs of six Lorenz systems.

### Irregular networks

One of the most interesting cases is making irregular networks because generate some behaviour that is not expected. It could be more complex if the elements of the irregular network are structurally different. This case is presented within the irregular Small-World topology and Tree topology. The first case is realized with identical chaotic systems and the second one with different chaotic systems.

#### Small-world topology

**Fig 9:**
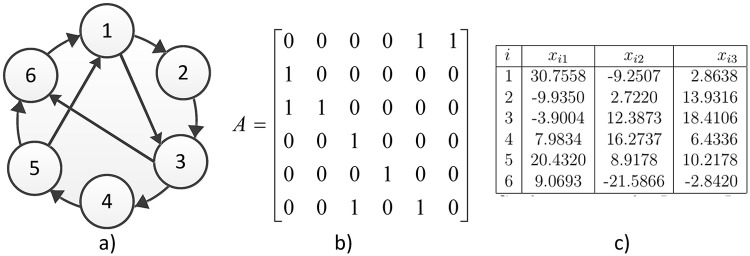
a) Small-World topology b) coupling matrix, c) random initial conditions.

**Fig 10:**
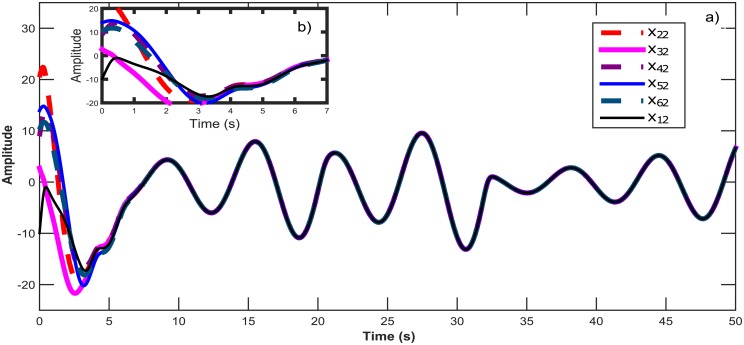
a) Synchronization graphics for six Rössler chaotic systems in a Small-World topology. b) Zoom in window showing the convergence in time for all *x*_*i*2_ states.

**Fig 11:**
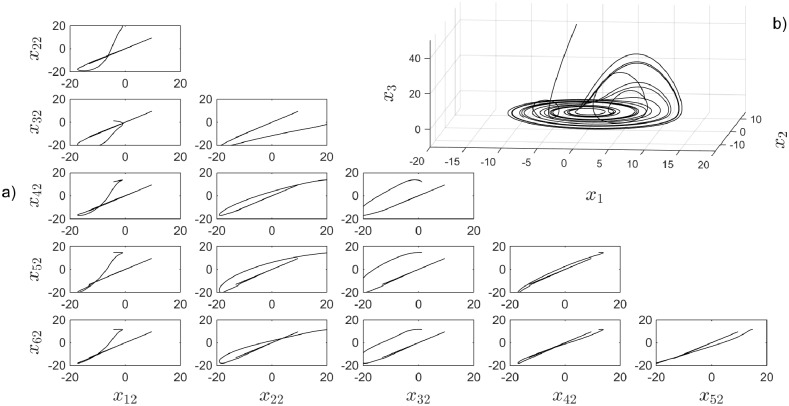
a) Synchronization graphics for six Rössler chaotic systems in a Small-World topology with an isolated node. b) Rössler chaotic attractor.

Small-world topology is a very used topology for the effectiveness to achieve synchronization. Six Rössler chaotic systems are synchronized when they are connected as shown in Fig 9 with the corresponding coupling matrix. Fig 10 shows the temporal output signals and the synchronization in a small time. Fig 11 presents the synchronization graphics for six Rössler chaotic systems in a Small-world topology.

#### Tree topology

**Fig 12:**
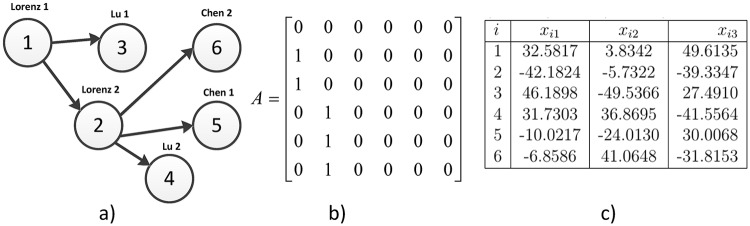
a) Tree topology with different chaotic systems, b) coupling matrix, c) random initial conditions.

**Fig 13:**
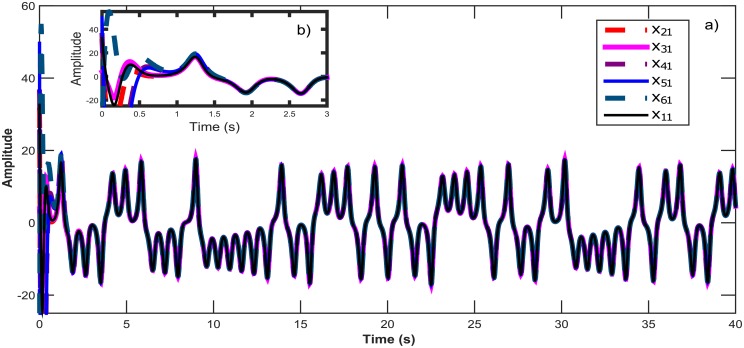
a) Output Synchronization for six nodes using three different chaotic systems in a Tree topology with two master systems. b) Zoom in window showing the convergence in time for all *x*_*i*1_ states.

**Fig 14:**
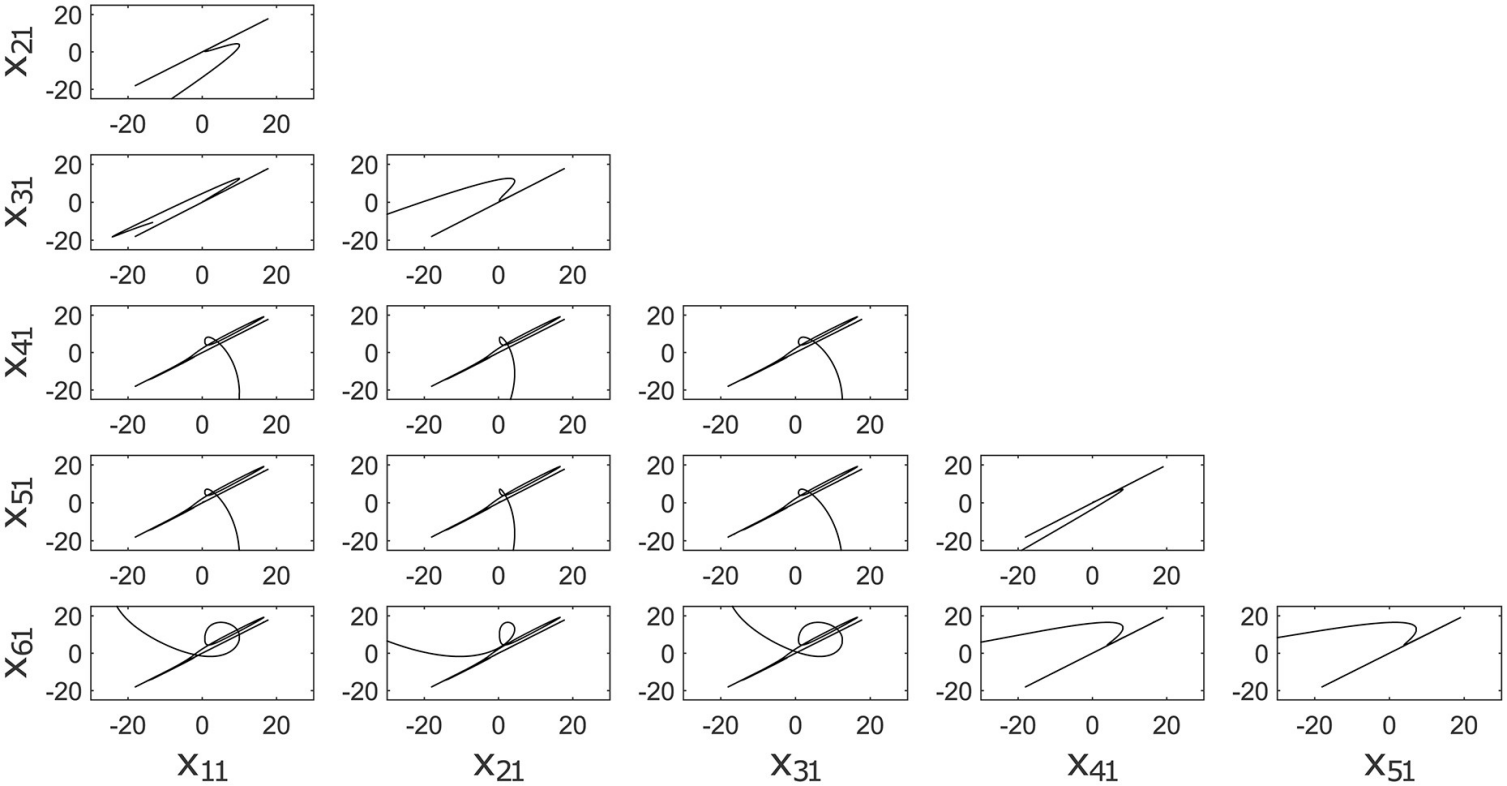
Output Synchronization graphics for different chaotic systems in a Tree topology.

**Fig 15:**
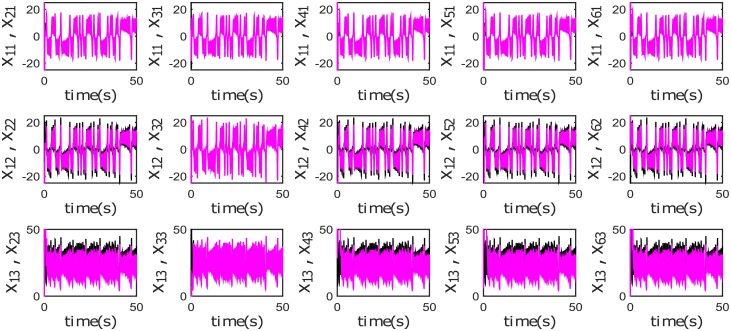
Output Synchronization, phase synchronization and complete synchronization for different chaotic systems in a Tree topology.

In this case, an irregular Tree topology is realized using an array presented in Fig 12 with its associated coupling matrix. Notice that it uses three different chaotic systems, two Lorenz systems, two Lü systems and two Chen systems. The main master is labeled as Lorenz 1 at the node 1. The node 2 contains the Lorenz 2 that is a slave system driven by Lorenz 1 and is another Master system. For non-identical chaotic systems, output synchronization if guaranteed in [11]. Then, the expected result is output synchronization. Fig 13 shows the temporal series for output synchronization for six nodes using three different chaotic systems in a Tree topology with two master systems. The output synchronization result is illustrated again in Fig 14 with the synchronization graphics. The complete and more interesting result is shown in Fig 15. All chaotic systems show output synchronization, whereas the other states show phase synchronization with respect to the main master node 1. Nevertheless, the states between both Chen systems and both Lü systems show complete synchronization in other new state in phase with the main master.

### Discussion

The MMC for network synchronization presented in this work is competitive with the most popular methodology used in [1–7, 14]. In that case, an error feedback between the signals to be synchronized is used. Synchronization time depends on the coupling gains *k*. On the other hand, in this work, synchronization time depends on the poles location. An adequate election on the poles location can achieve robust synchronization and, at the same time, an unforced control law. Nevertheless, some limitations were observed in the process: If Initial conditions are much bigger respect to the maxima amplitude of the temporal chaotic signal, the poles of the linearized auxiliary system need to be more negative from the complex axis of complex plane to achieve synchronization. This makes the MMC be increased, generating a forced control law. Other limitation is considered in Eq (5), where model matching problem is locally solvable if, and only if *r*_*i*_ ≤ *r*_*M*_. Nevertheless, it is possible to obtain output synchronization for systems with different order, if the systems hold condition (5). The main advantages for network synchronization using MMC are the following: It is a systematic procedure, it is robust, can be applied for nonidentical chaotic systems, and the initial conditions rank is bigger respect to other methodologies. They could be considered as disadvantages: Mathematical analysis for computing the control law is complex. When synchronization is more robust, a more forced control law is obtained. This is not desirable for physical implementation purpose, but it could be solved using hybrid systems (computer + physical implementation) [25]. This could be truth because the MMC does not increase the computation complexity, i.e., the computer time running the program depends only on the computer hardware and software.

## Conclusions

In this work, a synchronization scheme for complex networks of identical and nonidentical chaotic systems was presented. In particular, model-matching problem from nonlinear control theory was used. The results show complete synchronization for networks of identical systems and output synchronization for an irregular network of a class of non-identical systems. Although for non-identical systems, complete synchronization was achieved between Chen’s and Lü’s systems. The advantages over other cited approaches to synchronize networks of nonidentical chaotic systems are the following: this approach is a systematic procedure, it could use unidirectionally and bidirecctionally coupled systems, gains for the controller could be small, and synchronization network is obtained after a short transient behavior. Moreover, the proposed control law allows to chose any chaotic system as a model and more than one model. Numerical simulations were done. In future works, network layers and some applications for neural network communication systems will be studied. At the same time, some studies on the rank of the initial conditions and poles location could be analyzed.
